# Evaluation of Benzo[a]pyrene in Food from China by High-Performance Liquid Chromatography-Fluorescence Detection

**DOI:** 10.3390/ijerph9114159

**Published:** 2012-11-14

**Authors:** Yong-Hong Chen, En-Qin Xia, Xiang-Rong Xu, Sha Li, Wen-Hua Ling, Shan Wu, Gui-Fang Deng, Zhi-Fei Zou, Jing Zhou, Hua-Bin Li

**Affiliations:** 1 Guangdong Inspection and Quarantine Technology Center, Guangzhou 510623, China; Email: cathy66@21cn.com (Y.-H.C.); zfzhou@163.com (Z.-F.Z.); 2 School of Public Health, Guangdong Medical College, Dongguan 510234, China; Email: enqinxia@yahoo.com; 3 Key Laboratory of Marine Bio-resources Sustainable Utilization, South China Sea Institute of Oceanology, Chinese Academy of Sciences, Guangzhou 510301, China; 4 Guangdong Provincial Key Laboratory of Food, Nutrition and Health, Department of Nutrition, School of Public Health, Sun Yat-Sen University, Guangzhou 510080, China; Email: lisha0308@hotmail.com (S.L.); lingwh@mail.sysu.edu.cn (W.-H.L.); wushansw@sina.com (S.W.); misyfly@163.com (G.-F.D.); jingzhou@163.com (J.Z.)

**Keywords:** benzo[a]pyrene, pollutant, food analysis, food safety, public health

## Abstract

The occurrence and levels of benzo[a]pyrene in various heat-treated foods from China were evaluated by high-performance liquid chromatography-fluorescence detection. In a total of 119 samples, 105 were found to contain benzo[a]pyrene at levels of 0.03 to 19.75 µg/kg. The benzo[a]pyrene contents in 12 animal source foods were higher than the Chinese maximum permissible level in food (5 µg/kg) and the highest level was 19.75 µg/kg, nearly four times the maximum permissible level. The results revealed a widespread carinogenic public health risk from benzo[a]pyrene in heat-treated foods. The highest benzo[a]pyrene levels were found in animal source samples such as charcoal-grilled and smoked meats, especially pork, beef and sausage, while trace levels of benzo[a]pyrene were present in grain food. Charcoal-grilled vegetables were found to also contain certain levels of benzo[a]pyrene. This study provided new information on benzo[a]pyrene content of a variety of heat-treated foods from China.

## 1. Introduction

Research on food safety is attracting growing interests in the field of public health all over the World. Harmful compounds in food come from various sources, such as environmental pollution, food processing and illegal additives [1,2,3,4,5]. Besides coming from environmental pollution, polycyclic aromatic hydrocarbons (PAHs) have been found in protein-rich food products where they are generated during certain food processing procedures, such as smoking, heating (grilling, roasting) drying, or addition of smoke flavoring products [6,7,8,9,10,11,12,13,14,15,16,17]. Sixteen PAHs have displayed potentially genotoxic and carcinogenic effects on humans in many assays. The main mechanism of these negative health effects was found to involve their effects on enzymes, especially on the cytochrome P450 (CYP) family of enzymes, including CYP1A [18,19,20,21]. Research has also shown that PAHs could be transferred from their mothers to newborns and young infants, and cause brain and behavior toxicity [1,22]. Moreover, the diet may be the major source of exposure to PAHs for people who are non-tobacco-smokers and non-occupationally exposed. Benzo[a]pyrene (B[a]P), due the fact it is the most carcinogenic of the 16 PAHs and the PAH profiles occurring in food, is frequently used as a marker of the occurrence and effect of carcinogenic PAHs in food [23,24]. Lijinsky and Shubik [25] first reported the presence of B[a]P in charcoal broiled beef. Rey-Salgueiro and coworkers reported that the levels of total PAHs and benzo[a]pyrene in foods cooked by wood flames were 350 and 0.23 μg/kg, respectively [26]. The International Agency for Research on Cancer (IARC) has upgraded its overall evaluation of B[a]P from group 2B (possibly carcinogenic to humans) to 1 (carcinogenic to humans). The carcinogenic risk to public health of B[a]P in food has been widely considered by many government agencies and national authorities, and many countries have set limits to control the levels of B[a]P and thus reduce public exposure. 

Some methods of sample preparation and determination of PAH and B[a]P have been reported, which mainly involved in high-performance liquid chromatography with ultraviolet and fluorescence detection [27,28,29,30,31,32,33,34,35,36,37,38]. According to an IARC report in 1987, 7% of 10,000 food samples were found above the maximum admissible level for B[a]P, and detectable levels of B[a]P were found in 72 of the 95 food categories and in 47% of the samples tested. A limit value of 1.0 µg/kg was established by the European Union. Limited research found in the literature also showed high concentrations of B[a]P in foods. Terzi *et al*. [39] and Aygun *et al*. [40] reported that charcoal-grilled meat contained high concentrations of B[a]P, with average levels ranging from 5.7 ± 3.48 to 24.2 ± 0.84 μg/kg. This indicated that the public was exposed to B[a]P levels above the guideline value and it is quite necessary to carry out widespread and frequent monitoring surveys. However, no report on systematic surveillance of B[a]P level in food from China could be found in the World scientific literature. 

Guangzhou City, with its rapid economic growth, is an important industrial, cultural, foreign trade and communications center in south China. It is also one of the most important traffic nodes for businessmen travelling between China Mainland and Hong Kong, Macau, Taiwan, and many countries in the World as well. Therefore, there are people from many different regions or countries living in Guangzhou. The quality of food sold in Guangzhou not only relates to the health of Chinese people, but also the health of visiting guests from other countries. The foods prepared by smoking and grilling, especially charcoal-grilled meat, are widely consumed by the public due to its favored flavor. When the levels of B[a]P in some charcoal-grilled and smoked food samples from Guangzhou were evaluated in our laboratory, considerably high concentrations of B[a]P (>10 µg/kg) were found in some foods, which exceed the admissible level in food of China (5 µg/kg), and may be hazardous for public health. Therefore, this study has been carried out to evaluate the level of B[a]P in 119 selected foods to supply new information on B[a]P levels in a variety of foods from China.

## 2. Experimental Section

### 2.1. Chemicals

Benzo(a)pyrene, 1-dodecanol, 1-undecanol, *n*-hexadecane and 1,10-dichlorodecane were bought from Sigma (St. Louis, MO, USA). Acetonitrile, methanol, acetone and ethanol were of HPLC grade and were purchased from Merck (Darmstadt, Germany). Phosphoric acid and sodium chloride were of analytical grade, and bought from Tianjin Chemical Reagent Company (Tianjin, China). Deionized water was used throughout the experiments. 

### 2.2. Apparatus

The ultrasound-assisted extraction was carried out in a KQ-600E ultrasonic device (Changzhou Nuoji Instrument Company, Changzhou, China) with an ultrasound power of 600 W, heating power of 800 W, and frequency of 40 kHz, equipped with a digital timer and a temperature controller. 

### 2.3. Sample Treatment

Food samples were purchased from various markets around Guangzhou, China, and stored at 4 °C in a refrigerator. These foods are widely consumed in China, and representative of typical Chinese foods. About 5 g of the sample was accurately weighed into a 50 mL centrifuge tube, and then mixed with an appropriate amount of cooled NaOH solution (15 mL, 1 mol/L). Then the mixture was homogenized at 98 °C bath for 3–6 h. About 5 mL of ethanol was added to keep the mixture boiling. Dichloromethane-hexane (25:75, v/v, 20 mL) was added and the mixture immediately shaken on a Vortex mixer for 10 min. The sample was centrifuged at 3,500 g for 10 min and the organic phase was collected and filtered (filter pore size 0.22 µm, Beihualiming, Beijing, China). Extraction procedures were repeated three times. Then, extracts were combined and evaporated to dryness by rotary evaporation at 35 °C. The residue was redissolved in an appropriate amount of acetone, and the mixture was filtered. The volume of the solution is about 559.3 μL. Finally, the solution was subjected to dispersive liquid-liquid microextraction based on the solidification of floating organic droplet (DLLME-SFO) procedure, as described below.

### 2.4. DLLME-SFO Procedures

A 5 mL aliquot of water was transferred into a 10 mL glass tube equipped with a screw-cap, and a mixture of 559.3 μL of acetone extract and 79.9 μL of *n*-hexadecane was rapidly injected into water using a 2 mL syringe. The tube was irradiated by ultrasound in a water bath for 5 min at 36.3 °C. Then the tube was centrifuged at 3,500 g for 5 min, and the organic solvent droplets floated on the surface of the aqueous solution. The tube was transferred into 1–4 °C ice bath and the extraction solvent was solidified. After 10 min, the solidified solvent was transferred into a conical vial where it melted immediately at room temperature. Finally, 10 μL of the extract obtained was injected onto the HPLC column.

### 2.5. HPLC Analysis

**Figure 1:**
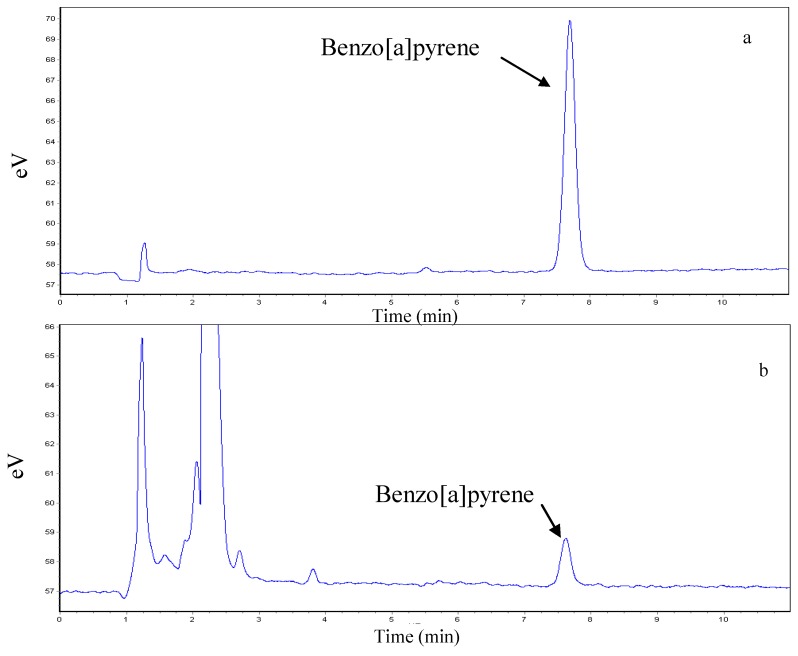
The chromatograms of benzo[a]pyrene in standard solution (**a**) and food sample (**b**).

A Waters (Milford, MA, USA) 1525 binary HPLC pump separation module equipped with a Symmetry C_18_ column (250 mm × 4.6 mm, 5 μm) was used. The mobile phase consisted of acetonitrile and water (9:1, v/v) at a flow-rate of 1.5 mL/min, and the column temperature was kept at 25 °C. The fluorescence detector was set at excitation wavelength of 361 nm and emission wavelength of 405 nm. The retention time of B[a]P was about 8.53 min. The peak area was used to calculate the amount of B[a]P from the standard curve. The typical chromatograms obtained from standard B[a]P and food sample are shown in Figure 1.

### 2.6. Statistical Analyses

The statistical package software version 7.1.3 (Stat Soft Inc., Tulsa, OK, USA) was used for experimental design analysis and data processing. Data for B[a]P levels of analysis of variance was performed to test the difference in B[a]P levels depending on food type.

## 3. Results and Discussion

### 3.1. Validation of the Analytical Method

Before investigation of the samples, the DLLME-SFO-HPLC-FLD method for B[a]P analysis was validated. The linearity of the calibration curve was tested using a series of standard solutions of B[a]P in acetone ranging from 0.2 to 100 µg/L. Furthermore, the recoveries of the proposed method were verified out by spiking several already analyzed samples with two levels (0.5 and 1.0 times) of standard solutions of B[a]P. The method showed a good linearity in concentrations ranging from 0.2 to 100 μg/L with a correlation coefficient of 0.9996. The limit of detection was 0.013 μg/kg based on a signal/noise ratio of 3:1 [41], which was far lower than the maximum levels fixed by current regulations. In addition, the limit of quantification (LOQ) was 0.055 μg/kg. The recovery values indicated the considerably high accuracy and precision of the proposed analytical procedure (ranging from 85.1% to 91.3% with 4.9~9.0% of RSD). In addition, the contents of B[a]P in several food samples were simultaneously determined by DLLME-SFO-HPLC-FLD in this study and the method based on the National Standards of Peoples Republic of China GB/T 5009.27-2003. No significant differences between the results of two methods were observed (*p* < 0.05). 

### 3.2. The Occurrence and Levels of B[a]P in Foods

**Figure 2:**
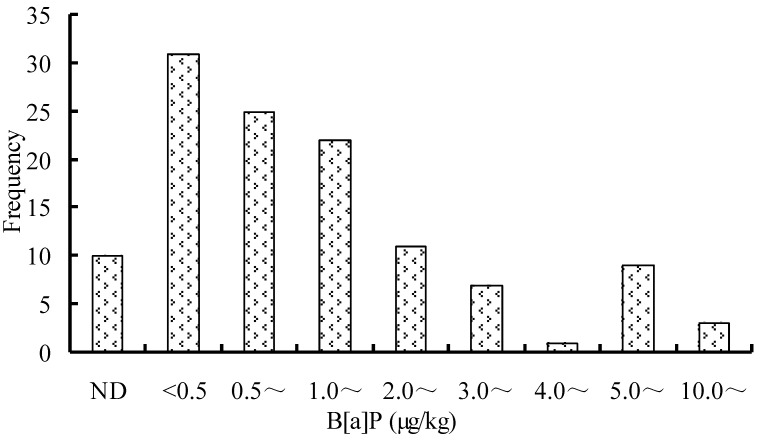
Frequency distribution of B[a]P contents in food.

The average, standard deviation and the ranges for the B[a]P contents in a total of 119 food samples classified in 12 categories and obtained from local markets in Guangzhou are summarized in Table 1. The frequency distribution of B[a]P contents in food is illustrated in Figure 2. One hundred and five out of the 119 samples showed levels of B[a]P with ranging from 0.03 to 19.75 µg/kg, which was above the LOD (Table 1). Fifty two out of 105 positive samples were found to contain B[a]P at levels lower than 1.0 µg/kg, and 22 samples out of 105 positive samples showed B[a]P contents ranging from 1.0 to 5.0 µg/kg. The levels of B[a]P in 12 of the positive samples ranged between 5.08 and 19.75 µg/kg. This means that 10.1% of the total samples contained B[a]P above the established Chinese food limit, where a maximal level of 5 µg/kg for B[a]P is recommended for food. This indicated that B[a]P was widely present in foods, and the highest level was near four times as high as the Chinese limit.

A wide variation was observed for B[a]P contents of the heat-treated foods between different food types. As seen from Table 1, the minimum contents of B[a]P were found in six positive samples out of nine cake dessert samples analyzed near the LOD, in which the highest contents of B[a]P were found even lower than 0.13 µg/kg. This trend is similar to that reported in a previous study, where Rey-Salgueiro *et al*. [26] reported that B[a]P did not generate until bread was heated to 300 °C, and the levels were lower than 0.5 µg/kg.

**Table 1:** The B[a]P levels in different kinds of foods.

Category of charcoal grilled samples	Samples tested	Sample positive	The level of B[a]P (µg/kg)			
			Mean	SD	Min	Max
Vegetable	8	8	0.48	0.22	0.26	0.86
Bean curd	7	5	0.92	1.04	0.52	3.27
Dried fruit	6	2	0.22	0.22	0.1	0.58
Cake dessert	9	6	0.03	0.04	0.03	0.13
Pork	11	11	2.01	2.34	0.24	7.97
Fish and shrimp	22	20	1.97	2.11	0.32	9.97
Sausage	15	15	4.25	5.8	0.23	19.75
Beef	10	10	2.66	3.59	0.19	9.29
Chicken	17	16	1.85	2.54	0.2	10.96
Mutton	4	4	0.62	0.7	0.1	1.61
Duck	7	5	1.68	2.03	0.91	5.97
Rabbit meat	1	1	1.64	-	-	-
Total	119	105	1.84	3.00	0.03	19.75

Furthermore, 15 among 21 samples of dried fruit, charcoal grilled vegetable and bean curd tested appeared to contain high levels of B[a]P compared to the cake dessert samples, and the average values of B[a]P were 0.22, 0.48, 0.92 µg/kg, respectively. This indicated that a certain carcinogenic risk of B[a]P for public health comes from charcoal-grilled vegetables and bean curd, through levels were found to be lower than the Chinese limit. 

**Figure 3:**
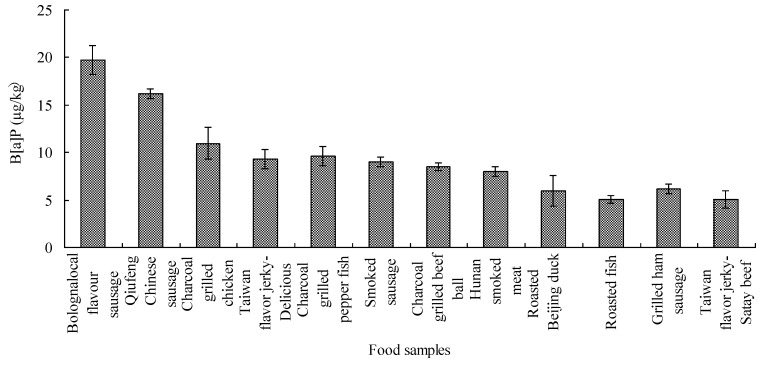
The B[a]P contents in 12 samples exceeding the Chinese limit.

Different animal meats prepared by heat-treatment, such as charcoal grilling, roasting or smoking, contained various levels of B[a]P, and the average contents increased as follows:, mutton, rabbit meat, duck, chicken, fish, shrimp, with average values of 0.62–1.97 µg/kg for the 46 positive samples out of 53 samples analyzed. In the 119 samples analyzed, all 36 samples containing the highest levels of B[a]P analyzed were detected in charcoal grilling or smoking samples, such as pork, beef, and sausage, with the highest average values of 2.01, 2.66 and 4.25 µg/kg, respectively. This might be because high temperatures (400–500 °C) and long heating times (15–20 min) were used for charcoal grilling animal product foods. Furthermore, all 12 samples containing B[a]P above 5 µg/kg (the Chinese limit) were heated-treated animal source foods. The B[a]P contents in 12 samples exceeding the Chinese limit are illustrated in Figure 3. Seen from this Figure, the B[a]P contents that were higher than 10 µg/kg were observed in three samples, including Bologna local flavor sausage, Qiufeng Chinese sausage, and charcoal grilled chicken, with contents of 19.57, 16.21 and 10.96 µg/kg, respectively. Nine heat-processed animal origin foods including Taiwan flavor beef jerky, charcoal grilled and roasted fish, smoked sausage, charcoal grilled beef ball, Hunan smoked meat, grilled ham sausage, roasted Beijing duck, were observed to contain B[a]P at concentrations from 5.08 to 9.29 µg/kg. The B[a]P in charcoal-grilled meat samples such as beef and chicken was at the same average level as seen in previous studies, that indicated B[a]P at concentrations of 3.16 and 2.44 µg/kg, respectively [42]. The results indicated that B[a]P was mainly present in heat-treated animal source foods, and a considerably heavy B[a]P pollution in animal source foods from local markets in Guangzhou.

From the results of the present study, it can be inferred that fat content might be an impacting factor for the generation of B[a]P in foods during food processing. Some studies have examined the effect of sample composition on B[a]P formation in meat samples. The results showed that the smoke induced by the fat during the heating procedures rises and penetrates into the meat [26,42]. This reason can explain why the smoked meat and sausage contained the highest levels of B[a]P ranging from 7.97 to 19.75 µg/kg in the present study. In addition, to test the effect of cooking method on the generation of B[a]P in food, mutton samples charcoal grilled to different degrees was analysed in the present study. The results showed that over-grilled meat might induce a nearly 10 times higher content of B[a]P compared to the slightly grilled samples, which agreed with a previous report [42]. Therefore, it indicated that the possible strategies to avoid the generation of B[a]P in food were preventing meat fat from dropping onto the fire or preventing the meat from directly contacting the heat source [26,42]. In Guangzhou, the heat-treated products, such as smoked, charcoal grilled and roasted food, are popular in local marketd due to the dietary customs, which are stimulated by the booming night-life. The present results indicated that the local public was exposed to a heavy carcinogenic risk from B[a]P in Guangzhou. This study supplied new data on B[a]P levels in a variety of foods from China, which should be very helpful for the public to avoid the exposure to high levels of the contaminant.

## 4. Conclusions

B[a]P was widely present in heat-treated foods obtained from local markets in Guangzhou. An 88.2% of a total of 119 samples contained B[a]P, among which the B[a]P contents in 12 animal source foods were higher than the Chinese maximum permissible level and the highest level was nearly four times as high as the Chinese food limit. The highest B[a]P levels were found in charcoal-grilled and smoked animal source samples, especially pork, beef and sausage, while trace levels of B[a]P were present in grains. Charcoal-grilled vegetables were found to contain certain levels of B[a]P as well. The present study provides important information on B[a]P in heat-treated food in China. This might help evaluate the presence and effects of contaminants in food, to assure food safety and to protect public health.
